# Ergonomic strain of robotic-assisted versus laparoscopic inguinal hernia repair (ESRALI)—a crossover trial

**DOI:** 10.1007/s00464-025-11676-7

**Published:** 2025-03-31

**Authors:** Alexandros Valorenzos, Kristian Als Nielsen, Per Helligsø, Michael Festersen Nielsen, Philip Wolfgang, Gert Frank Thomsen, Tina Dalager

**Affiliations:** 1 Department of General Surgery, University Hospital of Southern Denmark, Kresten Philipsens Vej 15, 6200 Aabenraa, Denmark; 2 Department of Regional Health Research, University of Southern Denmark, Odense, Denmark; 3 Department of Clinical Research, University Hospital of Southern Denmark, Aabenraa, Denmark; 4 Department of Sports Science and Clinical Biomechanics, Faculty of Health Sciences, University of Southern Denmark, Odense, Denmark; 5 Department of Occupational Medicine, University Hospital of Southern Denmark, Esbjerg, Denmark; 6 Department of Clinical Medicine, Faculty of Health Sciences, Aarhus University, Aarhus, Denmark

**Keywords:** Robotic-assisted laparoscopy, Laparoscopy, Inguinal hernia repair, Ergonomics, Surface electromyography, Kinematic analysis

## Abstract

**Background:**

Robotic-assisted and laparoscopic techniques are widely used for inguinal hernia repair. While robotic-assisted transabdominal preperitoneal (rTAPP) repair is believed to offer ergonomic advantages for surgeons, there is limited evidence comparing its ergonomic impact to conventional laparoscopic TAPP (cTAPP) repair. This study aims to assess the ergonomic strain on surgeons during these procedures using subjective and objective measures.

**Methods:**

This crossover observational study involved four experienced hernia surgeons who performed two procedures using each technique. Ergonomic strain was evaluated through self-reported perceived exertion (using the Borg scale), surface electromyography (sEMG) of select muscle groups, and posture analysis using rapid upper limb assessment (RULA) scores derived from kinematic data collected via Xsens Awinda. Statistical comparisons were conducted using the Wilcoxon rank-sum test, with adjustments for multiple comparisons.

**Results:**

Subjectively, surgeons perceived rTAPP as less physically demanding, with lower postoperative perceived exertion scores (median 1.5 vs. 3.0, *p* < 0.01). Objective measurements showed higher static muscle activity in the left erector spinae and median activity in the right trapezius during rTAPP (*p* = 0.016), but overall ergonomic strain, as indicated by RULA scores, was similar across modalities. Median RULA scores for both techniques were 3, and no significant differences were observed in work posture scores. Despite these findings, discomfort during cTAPP was more frequently reported, with surgeons citing the neck, shoulders, and lower back as affected areas.

**Conclusion:**

While rTAPP was subjectively perceived as less physically demanding, objective metrics did not corroborate these perceptions, showing comparable ergonomic strain between techniques. These findings highlight a complex relationship between subjective and objective ergonomic measures and suggest a need for further research, incorporating broader assessments of cognitive and physical loads, to optimize surgeon ergonomics in minimally invasive procedures.

**Graphical abstract:**

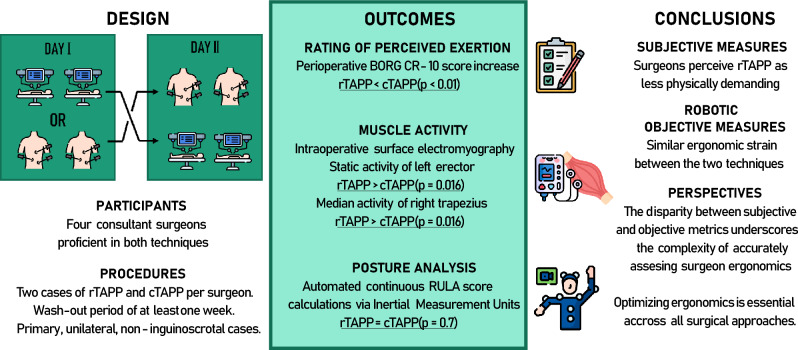

**Supplementary Information:**

The online version contains supplementary material available at 10.1007/s00464-025-11676-7.

Surgeons represent a profession with high rates of musculoskeletal pain [1]. Due to awkward and static work posture, surgeons face a demanding physical work environment, which in addition to raising mental demands, adds to the increased risk of developing muscular fatigue and pain. Moreover, literature shows that ergonomics and physical demands are critical factors in surgical performance, as they can affect surgeons’ comfort, precision, and risk of patient errors [2].

Inguinal hernia is a common surgical condition that affects millions of people worldwide, with a reported lifetime incidence of up to 27% in men and 3% in women [3]. Inguinal hernia repair is one of the most frequently performed surgical procedures in general surgery, and it can be performed using various techniques, such as open repair, laparoscopic repair, and robotic-assisted repair [4, 5].

Transabdominal preperitoneal (TAPP) inguinal hernia repair is one of the most commonly performed laparoscopic techniques for inguinal hernia repair. Laparoscopic approaches may be preferred over traditional open repair in certain patient groups due to advantages, such as reduced postoperative pain, lower rates of surgical site infections, earlier return to normal activities, improved cosmetic outcomes, and a slightly lower risk of chronic groin pain [6, 7].

Robotic-assisted inguinal hernia repair, though relatively new, has become widely adopted in surgical practice over the past decade [8]. This increasing adoption can partly be attributed to the perception that robotic-assisted surgery offers the advantages of laparoscopic repair, along with purported technical benefits, such as improved visualization, greater precision, and seemingly more ergonomic positioning for the surgeon and the assistant. The increased adoption has been accompanied by an expansion in studies demonstrating the feasibility of the procedure, its comparable safety profile, and, in some cases, superior clinical outcomes compared to conventional laparoscopic repair [5, 9–17]. On the other hand, the literature also suggests that it is more costly and time consuming compared to conventional laparoscopic repair [10, 15–19].

In terms of surgeon ergonomics, conventional laparoscopic surgery is characterized by repetitive motions, static work postures, and awkward positions, which are associated with high levels of musculoskeletal discomfort [20–22]. Robotic surgery is often believed to be ergonomically superior, as the surgeon operates seated at a console with armrests rather than standing beside the patient. However, comparative studies evaluating robotic and conventional laparoscopic procedures are heterogeneous, largely reliant on self-reported measures, and report conflicting conclusions regarding the ergonomic benefits of robotic surgery [1, 23–26].

In the field of inguinal hernia repair specifically, evidence is even more limited. The RIVAL trial used the rapid upper limb assessment (RULA) tool to compare conventional laparoscopic TAPP (cTAPP) and robotic-assisted TAPP (rTAPP) in terms of ergonomic strain on surgeons and concluded that the robotic approach did not offer ergonomic benefits [10]. However, a limitation of their study was that RULA scores were collected by observers and their conclusions may have been influenced by observer bias.

Understanding the differences in ergonomics between cTAPP and rTAPP is essential for optimizing surgical outcomes and minimizing surgeon injuries. Given the scarcity of data comparing these approaches within inguinal hernia repair, there is a need for more high-quality studies using objective measures.

The aim of this study is to compare cTAPP and rTAPP for inguinal hernia repair in terms of surgeon ergonomics during surgery. We hypothesize that rTAPP reduces ergonomic strain for surgeons compared to cTAPP.

## Materials and methods

### Study design and data collection

This study adheres to the STROBE Statement for observational studies. It was designed as a crossover trial and conducted at the Department of General Surgery, University Hospital of Southern Denmark, from November 2023 to June 2024. The processing of personal data is notified to and approved by the Region of Southern Denmark and listed in the internal record (Reg No. 25/5557) cf. Art 30 of The EU General Data Protection Regulation. According to the guidelines from the National Committee on Health Research Ethics, a project must have a health scientific purpose and involve an intervention to fall within the committee’s reporting framework. Due to their observational nature, the trial-related procedures did not qualify as an intervention under the Committee Act, and therefore, the research project did not require reporting to the committee system. A waiver was obtained from the Regional Health Ethics Committee (Case No. S-20252000-20). The study was retrospectively registered in the Open Science Framework (OSF) registry (identifier: Y9ECH) and conducted according to an a priori protocol developed before the initiation of the research. The protocol outlined the study design, participant eligibility criteria, outcomes, and planned statistical analyses. It is available from the corresponding author upon reasonable request.

**Fig. 1:**
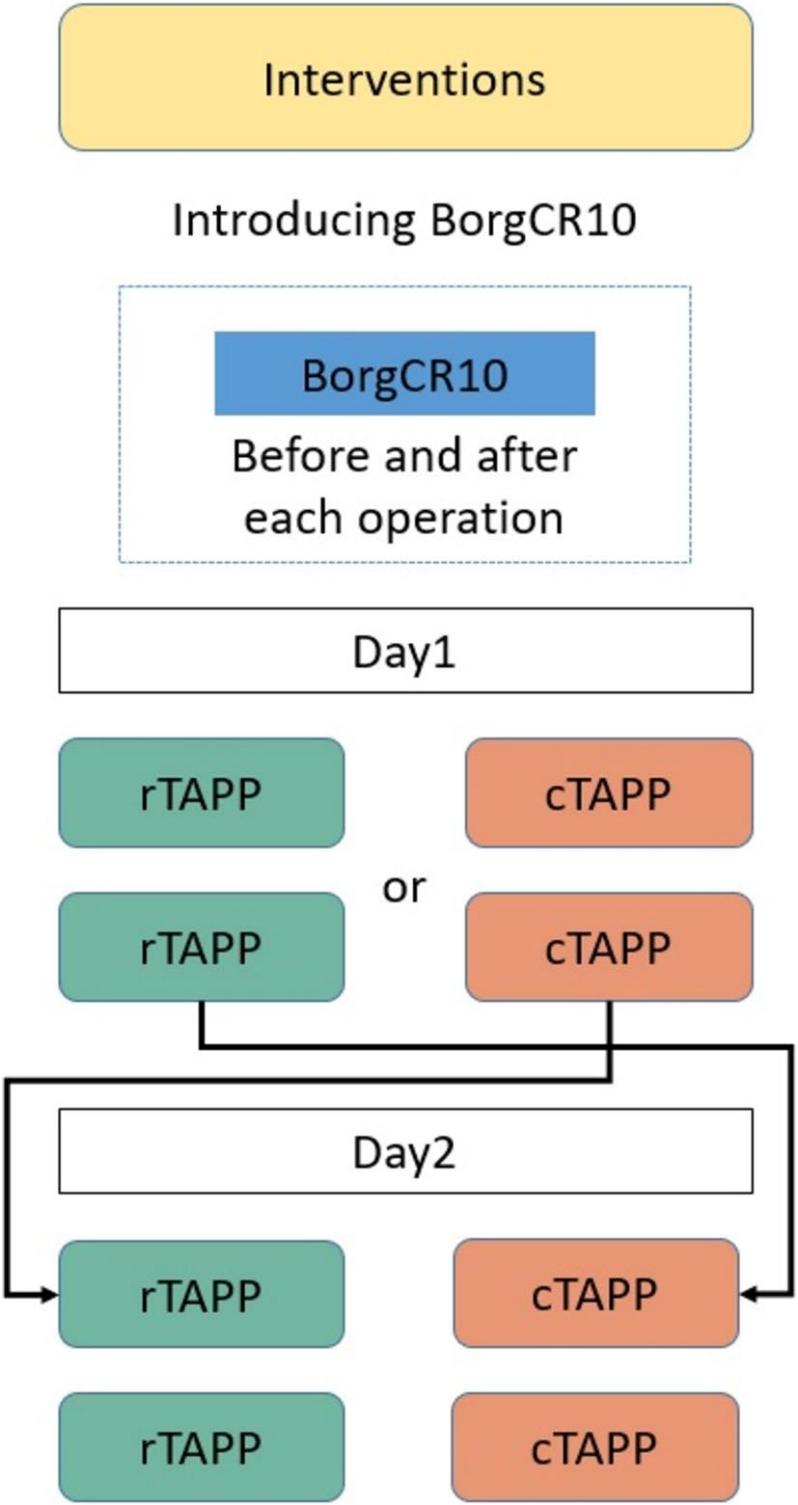
Study flowchart

Eligible participants were surgeons proficient in both procedures: rTAPP and cTAPP inguinal hernia repair. Four experienced hernia surgeons, each having performed over 100 procedures of both modalities prior to trial participation, and provided informed consent before enrolling in the study. Each participating surgeon performed two procedures of each surgical modality as part of the study. On their first assigned study day, the surgeon performed two consecutive procedures of one modality (either rTAPP or cTAPP) as the first surgeries of their workday. On their second assigned study day, scheduled after a washout period of at least one week, the surgeon performed two consecutive procedures of the alternate modality (Fig. 1).

Data collection included a short questionnaire, surface electromyography (sEMG) recordings of muscle activity, kinematic recordings of work posture during surgery, and self-reported assessment of physical exertion before and after each surgical procedure.

### Description of surgical procedures

All procedures included in this study were primary, unilateral, non-inguinoscrotal inguinal hernia repairs. To minimize potential confounding factors related to patient characteristics, procedures were selected to include cases with comparable surgical complexity and patient profiles, including body composition within clinically acceptable ranges.

The cTAPP procedure was performed with the patient positioned supine under general anesthesia. A 12 mm laparoscope port was placed at the umbilicus, and two 5 mm working ports were inserted in the same transverse plane as the umbilical port, 6–8 cm laterally on each side. CO₂ insufflation created a pneumoperitoneum at a pressure of 12 mmHg. The procedure utilized an Olympus ENDOEYE™ 3D 10 mm laparoscope. All surgeons used the same instruments, including Microline ReNew™ XR 34 cm Ratcheted Laparoscopic Handpieces with interchangeable instrument tips (scissors and graspers) and an Aesculap® Duogrip® TC straight needle holder. Electrocautery was performed via foot pedals. The peritoneum was incised, the hernia sac was dissected and reduced, and a ProGrip™ laparoscopic self-fixating mesh was placed over the defect. The peritoneum was closed over the mesh with barbed absorbable sutures (V-Loc™ wound closure device). Finally, the ports were removed, and the incisions were closed in layers, with the skin sutured using absorbable materials.

In the operating room, monitors were mounted on a suspension arm system, which allowed surgeons to position the monitors as they preferred. The height of the operating table was adjusted to each surgeon’s individual preferences. To ensure that ergonomic outcomes were not influenced, surgeons were not given any instructions regarding monitor placement, table height, or their positioning during the procedure.

The rTAPP procedure followed a similar technique but utilized the Da Vinci Xi Robotic Surgical System. Three 8 mm robotic ports were positioned in the same anatomic locations, and the robot was docked with its arms aligned to these ports. The surgeon operated remotely from the console, using the robotic arms to perform the steps described above. Surgeons were not instructed on how to adjust their seats, console armrests, or other ergonomic settings at the console, ensuring that their preferences were not influenced during the procedure.

### Questionnaire

The self-administered questionnaire collected data on demographics, work history, self-perceived work-related physical strain, musculoskeletal discomfort during surgery, strategies to alleviate discomfort, self-rated general health, and physical activity levels over the past year. An English version of the questionnaire is provided in Supplementary Material 1.

### Muscle activity

**Fig. 2:**
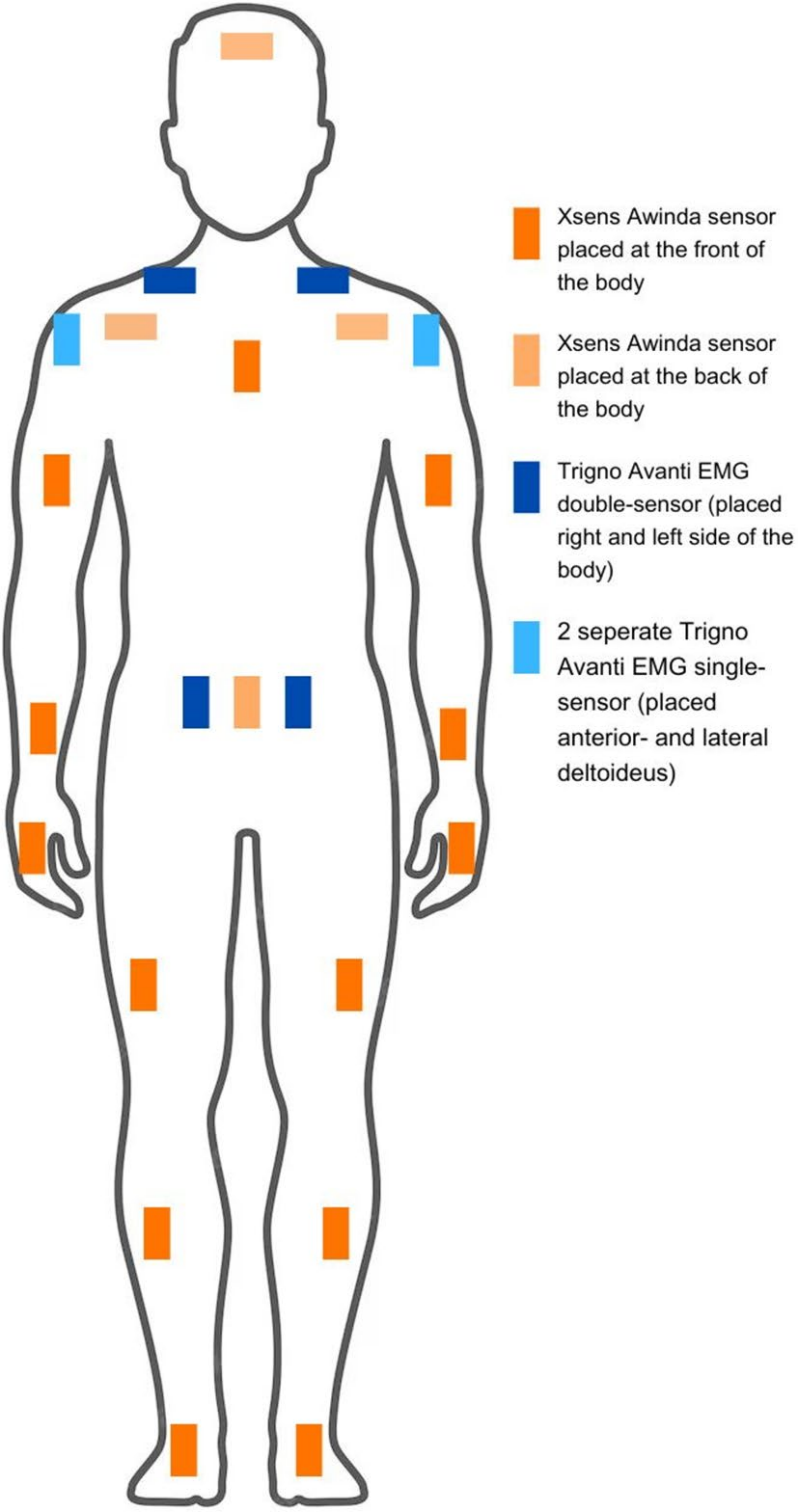
Placement of sensors for kinematic and surface electromyography data collection

Muscle activity was measured using the TRIGNO™ Avanti wireless EMG system (DELSYS), which accommodates up to 16 sensors and operates at a sampling rate of 1024 Hz. EMG data were analyzed using MATLAB software. sEMG sensors were placed bilaterally on the anterior deltoid, middle deltoid, upper trapezius, and erector spinae muscles (Fig. 2). Placement adhered to the SENIAM guidelines for sEMG sensor positioning [27]. Preparation of the sensor placement sites included shaving, scrubbing with an abrasive paste, and cleaning with 70% alcohol.

To normalize muscle activity, EMG signals were expressed as a percentage of the maximum voluntary contraction (%MVC). MVCs were determined for each muscle group through three tests per muscle, with 30 s of recovery between tests. A light warm-up was performed before the MVC tests.

### Work posture

Kinematic data on work posture were collected using the Xsens Awinda system (Movella, Enschede, Netherlands). The system includes 17 Inertial Measurement Units (IMUs) distributed across the body (Fig. 2). Each IMU contains a 3D accelerometer, 3D gyroscope, and 3D magnetometer. These components generate data that are merged by a proprietary algorithm, providing information on angular velocity, rotation, acceleration, directional changes, and orientation. The IMUs communicate wirelessly with the Awinda station, a dedicated hub that ensures time synchronization across sensors and connects to a host PC [28].

Prior to each surgical procedure, the surgeon donned a measuring suit with the IMUs securely attached, and the Awinda system was calibrated to establish a baseline for each participant. Work posture was evaluated using the rapid upper limb assessment (RULA) tool [29]. RULA is a standardized screening method for assessing body posture and estimating total body strain. It generates a “RULA grand score” on a scale from 1 to 7, which determines the urgency of ergonomic intervention. Scores 1–2 indicate acceptable posture if not maintained, 3–4 suggest that further investigation may be needed and that changes might be advised, 5–6 indicates the need for further investigation and changes soon, while a score of 7 suggests that investigation and changes are required immediately.

For this study, Xsens data were exported in mvnx format and processed in MATLAB (R2021a; MathWorks, Natick, Massachusetts, USA) using a Python script provided by Movella to estimate functional RULA grand scores. To automate the calculation of RULA grand scores, we established joint angle thresholds (Table 1) that serve as specific cutoff points for those RULA steps not originally defined by precise angle criteria. This approach ensures that every scoring step can be consistently and objectively quantified.

**Table 1:** Joint angle thresholds used to determine RULA score adjustments

Action	Cutoff point	Points added
Raising of shoulder	≥ 5°	1
Abduction of upper arm	≥ 30°	1
Arms crossing midline or the lateral border of the body	Arm working across midline	1
	Arm working lateral to the body	1
Radio-ulnar deviation of wrist	Radial deviation ≥ 15°	1
	Ulnar deviation ≥ 25°	1
Rotation of wrist (pronation or supination)	≥ 20° in any direction	1
Rotation of neck	≥ 30° in any direction	1
Lateral flexion of neck	≥ 15° in any direction	1
Rotation of trunk	≥ 30° in any direction	1
Lateral flexion of trunk	≥ 15° in any direction	1

### Perceived physical exertion

The perceived physical exertion (RPE) was assessed using the ten-grade Borg Scale [30]. Surgeons subjectively reported their RPE, which reflects their perceived level of physical stress and strain. The scale ranges from 0 (indicating no exertion at all) to 10 (maximal level of exertion). Measurements were recorded before and immediately after each surgical procedure.

### Statistical analysis

Descriptive statistics were employed to present the results of the questionnaire.

For RPE, medians and ranges were determined for the preoperative and postoperative Borg scale ratings for each surgical modality. Differences between rTAPP and cTAPP were analyzed using the Wilcoxon rank-sum test, a non-parametric test suitable for ordinal data. A significance level of *p* < 0.05 was applied.

For muscle activity, the amplitude probability density function (APDF) was used to compare %MVC values across surgical modalities and muscle groups. APDF analysis quantified static muscle activity (10th percentile), median muscle activity (50th percentile), and peak muscle activity (90th percentile). The Shapiro–Wilk test was used to assess the normality of %MVC distributions, and since the data were not normally distributed, the Wilcoxon rank-sum test was employed. Due to multiple comparisons (eight muscles across three levels of muscle activity), a Bonferroni correction was applied, adjusting the significance level to a corrected alpha of 0.017.

For work posture, the final RULA score for each procedure was calculated as the median of the RULA grand scores across all frames in the complete time series. Additionally, a relative RULA score distribution was then determined by calculating the relative frequency of each RULA score value. Differences in the final RULA scores and the relative distributions of RULA scores between cTAPP and rTAPP were analyzed using the Wilcoxon rank-sum test, because the data were of ordinal nature and not normally distributed, as determined by the Shapiro–Wilk test. For the final RULA scores, the significance level was set at *p* < 0.05. For the relative RULA score distributions, a Bonferroni correction was applied to account for the four possible RULA score ergonomic significance levels, setting the corrected alpha level at 0.0125. Statistical analysis was performed using R and results were considered statistically significant if the *p* value was below the respective threshold.

## Results

### Baseline surgeon characteristics

The four participating surgeons were all male and right handed, with a median age of 58 years (range 50–58). Their median height was 185 cm (range 178–193) and their median weight was 89 kg (range 77–102). Glove sizes had a median of 7.5 (range 7.5–8),

All surgeons were highly experienced, with a median of 26.5 years in practice (range 20–33). Their weekly working hours were 53.5 (range 42–60). The number of operations performed per week as the primary surgeon had a median of 9 (range 4–15), while the number of operations as an assistant had a median of 2.5 (range 2–5).

Two surgeons rated their general health as “Good” (50%), while the other two rated it as “Very Good” (50%). Regarding physical activity levels over the past year, one surgeon reported being almost entirely inactive (25%), one engaged in light physical activity for 2–4 h per week (25%), and two engaged in either more than 4 h per week of light physical activity or 2–4 h of strenuous physical activity (50%).

Self-perceived physical strain during work, rated on a numeric scale from 0 to 10, had a median of 4.5 (range 2–6).

Three out of four surgeons (75%) reported experiencing musculoskeletal discomfort “often” during conventional laparoscopy, while one (25%) reported experiencing it “sometimes.” The most commonly affected body regions during conventional laparoscopy were the shoulders (100%), neck (75%), and lower back (75%), with additional reports of upper back (25%), elbow (25%), and wrist/hand discomfort (25%). In contrast, discomfort during robotic-assisted laparoscopy was reported as “rarely” by three surgeons (75%) and “sometimes” by one (25%). The affected body regions were more limited, with discomfort primarily reported in the wrists/hands (75%) and neck (50%).

To manage discomfort, all surgeons reported changing posture during surgery as a primary strategy (100%). Additionally, one surgeon (25%) used micro-breaks, while another (25%) reported using painkillers to alleviate musculoskeletal discomfort.

### Perceived physical exertion

Perceived physical exertion before and after each surgical procedure is summarized in Table 2. The median preoperative RPE was 0.5 (range 0.5–1.5) for both cTAPP and rTAPP. Postoperatively, RPE was significantly higher for cTAPP, with a median of 3.0 (range 2.0–3.5), compared to rTAPP, which had a median of 1.5 (range 0.5–2.5) (*p* = 0.00136).

**Table 2:** Pre- and postoperative Borg rating for each procedure

Surgeon ID	Surgery type	Preoperative value	Postoperative value	Increase in value
Surgeon 1	cTAPP	0.5	2.0	1.5
		0.5	3.0	2.5
	rTAPP	0.5	0.5	0
		0.5	0.5	0
Surgeon 2	cTAPP	0.5	3.0	2.5
		0.5	3.0	2.5
	rTAPP	0.5	2.0	1.5
		0.5	1.5	1.0
Surgeon 3	cTAPP	0.5	3.0	2.5
		0.5	3.5	3.0
	rTAPP	0.5	1.5	1.0
		0.5	1.5	1.0
Surgeon 4	cTAPP	1.5	3.0	1.5
		1.5	3.0	1.5
	rTAPP	1.5	2.0	0.5
		1.5	2.5	1.0

*rTAPP* robotic-assisted transabdominal preperitoneal inguinal hernia repair, *cTAPP* conventional laparoscopic transabdominal preperitoneal inguinal hernia repair

### Muscle activity

**Fig. 3:**
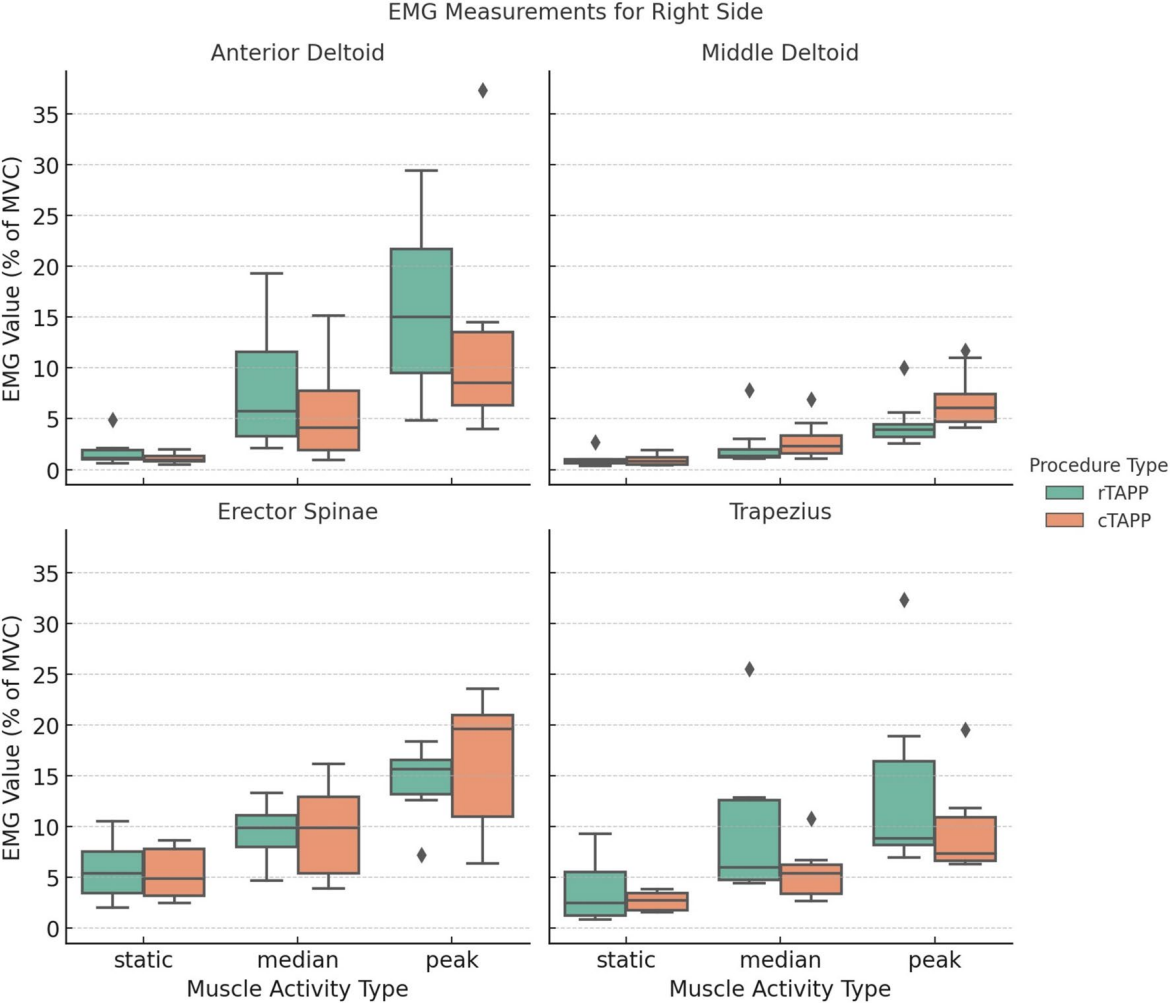
Static, median, and peak muscle activity for the right side

**Fig. 4:**
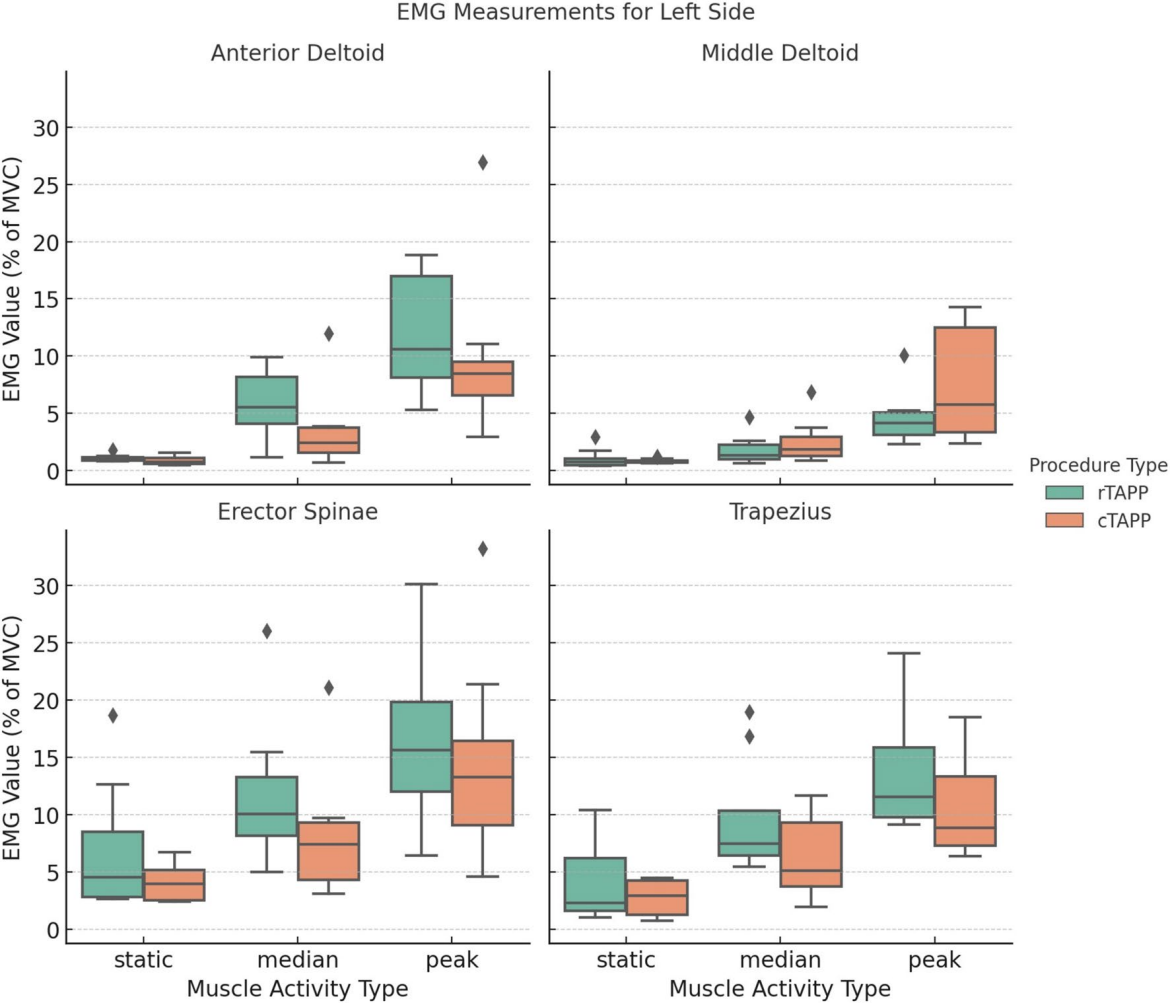
Static, median, and peak muscle activity for the left side

Figures 3 and 4 present the static, median, and peak levels of muscle activity across all recorded muscles. Detailed statistical results are provided in Supplementary Material 2. The erector spinae muscles consistently exhibited the highest levels of muscle activity, regardless of surgical modality. A statistically significant difference was observed in the static muscle activity of the left erector spinae between rTAPP and cTAPP after applying the Bonferroni correction (mean ± SD: 7.0 ± 5.8% MVC for rTAPP vs. 4.1 ± 1.6% MVC for cTAPP, *p* = 0.016). Similarly, the right upper trapezius muscle demonstrated a significant difference in median muscle activity between rTAPP and cTAPP (mean ± SD: 9.6 ± 7.3% MVC for rTAPP vs. 5.4 ± 2.6% MVC for cTAPP, *p* = 0.016). No other significant differences were found between the surgical modalities.

Across all muscles, static muscle activity ranged between 1.0–7.0% MVC for rTAPP and 0.8–5.3% MVC for cTAPP. Median muscle activity ranged between 1.8–11.7% MVC for rTAPP and 2.5–9.7% MVC for cTAPP. Peak muscle activity ranged between 4.6–16.0% MVC for rTAPP and 6.8–16.5% MVC for cTAPP.

### Work posture

**Fig. 5:**
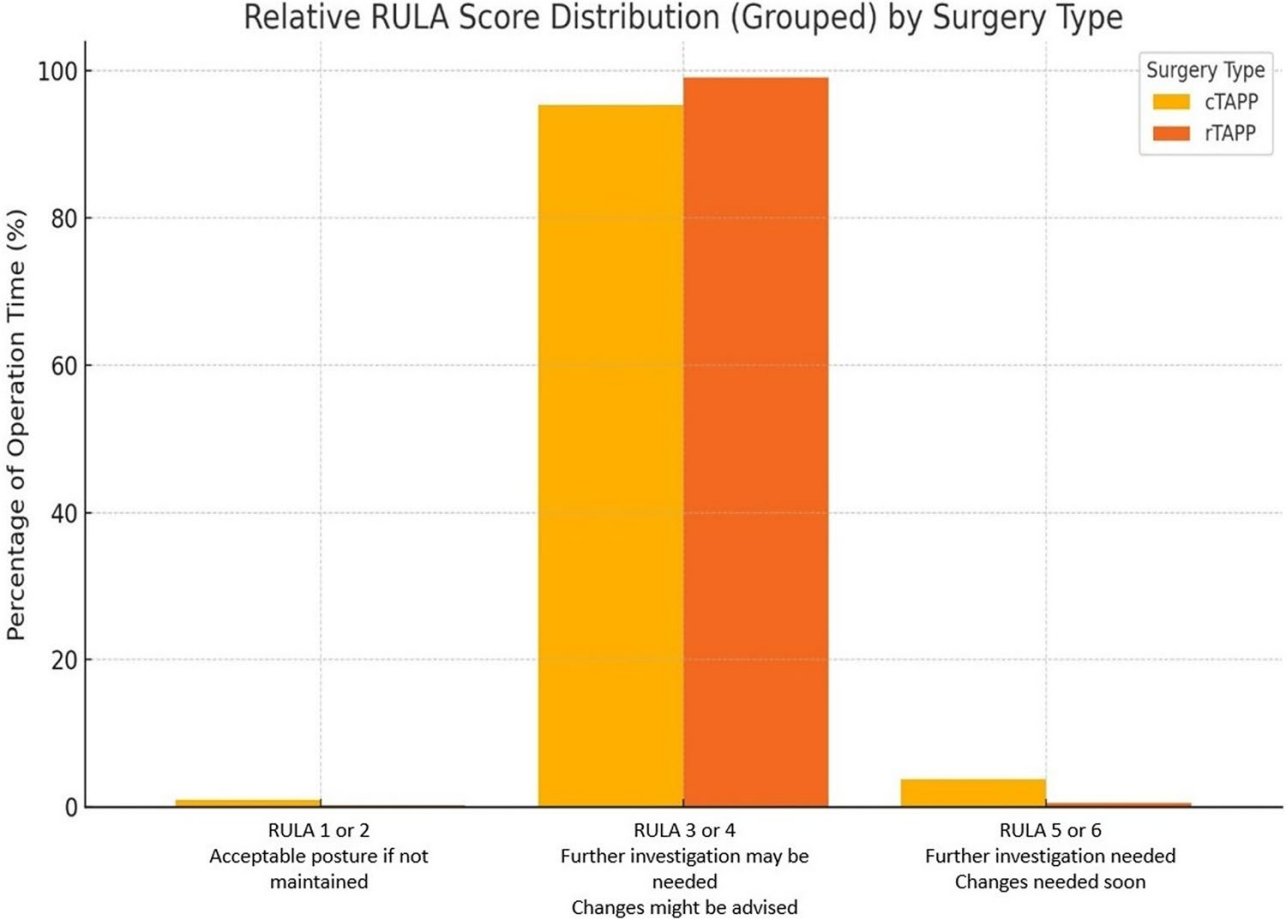
Relative RULA score distribution

Scores exceeding 4 were recorded for a larger proportion of the procedure during cTAPP than rTAPP (3.724% vs. 0.576%), although this difference was not statistically significant (*p* = 0.4621). The relative RULA score distribution otherwise varied between the two procedures (Fig. 5). Surgeons scored a RULA score of 1 or 2 for a larger portion of the procedure when performing cTAPP compared to rTAPP (0.935% vs. 0.206%; *p* = 0.0008). Conversely, surgeons scored RULA values of 3 or 4 for a smaller portion of the procedure during cTAPP compared to rTAPP (95.339% vs. 99.056%; *p* = 0.0157), but this difference did not reach statistical significance after applying the Bonferroni correction.

No significant difference was observed in the median RULA scores between rTAPP and cTAPP procedures (*p* = 0.674). The median RULA score for all procedures was 3, except for one rTAPP case, which had a median score of 4.

## Discussion

Our findings suggest that surgeons subjectively perceive rTAPP as less physically demanding than cTAPP, as reflected in lower RPE scores. This result aligns with previous literature reporting that surgeons often perceive robotic surgery as ergonomically superior to laparoscopic surgery [1]. However, the objective measurements of muscle activation and posture analysis did not support this subjective perception. Our results showed no significant overall differences in muscle activity or final RULA scores between the two modalities, aside from higher static activation in the left erector spinae and median activation in the right trapezius during rTAPP. Similarly, a systematic review by Shugaba et al. highlighted discrepancies between perceived and measured ergonomic strain, with increased trapezius activity observed during robotic surgery [31]. Interestingly, their findings also noted reduced activation in other muscles, such as the erector spinae, contrasting with our results. These inconsistencies across studies underscore the complexity of objectively assessing ergonomic strain in robotic versus laparoscopic surgery.

The discrepancy between subjective perceptions and objective findings may suggest that our measurements are not capturing the full spectrum of the ergonomic strain of surgery or that surgeons’ perceptions are influenced by factors beyond physical exertion alone. If rTAPP is not objectively more ergonomic, as our findings suggest, it raises questions about surgeon bias. Surgeons may report less discomfort with robotic surgery due to perceived technological superiority or psychological factors. For example, Norasi et al. reported that robotic surgeons were less likely to report discomfort, even when experiencing similar physical strain as laparoscopic or open surgeons [32]. This suggests that perceptions of comfort may be influenced by subjective expectations rather than actual physical demands. Additionally, stress or frustration during surgery could manifest as physical discomfort. Previous research has demonstrated that cognitive demands significantly influence ergonomic strain experienced by surgeons during both robotic and laparoscopic surgery, further complicating the relationship between perception and objective measures [23]. Yet, we did not directly measure mental or cognitive loads in this study and these elements could thus represent unmeasured confounders.

The objective measurements in our study, including sEMG and motion analysis, are well-established methods in ergonomic research [33]. For posture evaluation, we employed the widely used RULA tool, which is particularly suited for assessing upper-body intensive tasks, such as those performed during surgery. RULA is an observational risk assessment tool where scores are typically recorded by trained observers who document work processes manually. However, a limitation of this approach is its inability to assess the full range of occupational demands over an entire work process. As a result, it often focuses on postures that are most frequent, accounting for more than 10–15% of a task, and those considered most harmful. By integrating high-frequency kinematic data collection through the Xsens system, our study provides a more objective and comprehensive analysis of surgeons’ working conditions, addressing some of RULA’s observational limitations and enhancing the study’s strength.

However, other studies have utilized the rapid entire body assessment (REBA) tool instead. These studies, particularly in colorectal, bariatric, and urological procedures, have reported significantly lower REBA scores for robotic-assisted procedures compared to conventional laparoscopy [34–36]. While REBA is effective for evaluating tasks involving dynamic posture changes or heavy lifting, it is less detailed when analyzing sustained upper-body postures, which are critical in surgical practice. In contrast, RULA is better suited for assessing the static and sustained postures typical during surgery [33]. This difference in the scope of these tools may partly explain why REBA and RULA can yield differing ergonomic assessments between robotic and laparoscopic procedures.

In regard to muscle activity, we compared the two procedures using sEMG. We selected muscle groups that we anticipated would be most strained, following consultations with surgeons. Our findings of higher median trapezius activation in rTAPP are consistent with reports of elevated trapezius activity during simulated tasks performed with the robotic platform compared to conventional laparoscopy [23, 26, 37].

One potential explanation is the suboptimal adjustment of robotic armrests as many surgeons fail to adjust the armrests to the correct height, leading to increased shoulder/upper back strain [38].

On the other hand, both the aforementioned studies and others have reported reduced muscle activation in other muscle groups, such as the biceps, triceps, deltoideus, flexor carpi ulnaris, and flexor radialis, and during robotic procedures compared to laparoscopic approaches [39]. This raises the question of whether the higher perceived strain in cTAPP may be due to greater activation in muscle groups not included in our study.

Another limitation of our study is the small sample size, which may have reduced our ability to detect clinically significant differences. Larger sample sizes are critical for increasing statistical power and enhancing the reliability of findings. Furthermore, all participating surgeons in this study were male. While this reflects the demographics of the surgeons performing these procedures at our institution, it may limit the generalizability of the findings to female surgeons. Future studies should aim to include surgeons of different genders to better understand potential differences in ergonomic outcomes. However, each surgeon acted as their own control, performing two procedures of each modality, thereby reducing the risk of confounding. Additionally, unlike simulation-based studies, our measurements were conducted during live surgeries, which increases clinical relevance and generalizability but also introduces logistical challenges. Such constraints are common in similar research due to limited resources and time.

Interestingly, surgeons often fail to optimize their ergonomics, regardless of the type of surgery. Studies have shown that many surgeons do not utilize ergonomic aids, such as armrests, during robotic procedures [40]. However, Rodriguez et al. reported that with increasing experience, surgeons exhibited better ergonomic postures during robotic surgeries, indicating that proficiency with the robotic platform plays a crucial role in ergonomic optimization [26]. Furthermore, a feasibility study on ergonomic training for robotic surgery showed that structured training programs can improve surgeons’ awareness and use of ergonomic techniques [38]. This underscores the importance of ergonomic education and training to help surgeons fully benefit from robotic systems.

## Conclusion

In conclusion, while surgeons subjectively perceive robotic-assisted TAPP as less physically demanding than conventional laparoscopic TAPP, our objective measurements did not validate this perception. Although certain muscle groups, such as the trapezius and erector spinae, demonstrated higher activation in robotic surgeries, the overall ergonomic strain between the two modalities remained similar. This highlights the complexity of quantifying ergonomic strain, as it involves multiple factors, including physical, cognitive, and psychological elements. The discrepancy between subjective reports and objective data suggests that further research is needed, incorporating both physical and mental load assessments. Additionally, optimizing ergonomics through targeted training and better use of available aids is crucial for enhancing surgeon well-being across all surgical approaches.

## Supplementary Information

Below is the link to the electronic supplementary material.

Supplementary file1 (DOCX 72 KB)

Supplementary file2 (DOCX 14 KB)
