# Supplementary material for: Ergonomic strain of robotic-assisted versus laparoscopic inguinal hernia repair (ESRALI)—a crossover trial
Source: Surg Endosc. 2025 Mar 31;39(5):3095–105. doi: 10.1007/s00464-025-11676-7 (PMC12041149; doi:10.1007/s00464-025-11676-7)
Supplement: Supplementary file 1 — Supplementary file1 (DOCX 72 KB) [file 464_2025_11676_MOESM1_ESM.docx]

***S1 (English translation of the baseline questionnaire)***

**Would you like to participate in this survey?**

You consent to the following:
"I have received written information and know enough about the purpose, methods, benefits, and risks of participating to decide to participate. I know that participation is voluntary, and that I can withdraw my consent at any time (valid until publication, after which withdrawal is no longer possible)."

Yes, I consent to participate in the survey.

No, I do not wish to participate in the survey.

**What is your gender?**

Male

Female

Other

**What year were you born?** (e.g., 1980)

**How tall are you?** (cm)

**What is your weight?** (kg)

**How would you rate your overall health?**

Excellent

Very good

Good

Fair

Poor

**Please indicate your level of physical activity during leisure time (including transportation to and from work) within the last year (select one):**

Almost entirely physically inactive or lightly active for less than 2 hours per week

Light physical activity for 2–4 hours per week, e.g., walking, cycling, light gardening, light exercise

Light physical activity for more than 4 hours per week or strenuous activity for 2–4 hours per week, e.g., brisk walking, cycling, heavy gardening, intense exercise

Strenuous physical activity for more than 4 hours per week or regular intense training and competitions multiple times per week

**How long have you worked as a surgeon?**

Number of years: ______

Number of months: ______

**How many surgeries do you perform on average in a week as the primary surgeon?**

**How many surgeries do you perform on average in a week as an assistant surgeon?**

**How many hours do you work on average per week?**

Total working hours: ______

Hours spent operating: ______

**How physically demanding do you perceive your current job to be?**
Scale: 0 (Not demanding) – 10 (Extremely demanding)

0

1

2

3

4

5

6

7

8

9

10

**Do you experience musculoskeletal discomfort when performing surgery?**

Conventional laparoscopy

Yes, every time

Yes, often

Yes, sometimes

Rarely

No

Robot-assisted laparoscopy

Yes, every time

Yes, often

Yes, sometimes

Rarely

No

**If you answer "No" to musculoskeletal discomfort, you are finished with this questionnaire.**

**Please indicate where you primarily experience physical discomfort when performing surgery using conventional laparoscopy:**

Neck

Upper back

Lower back

Shoulders

Elbow

Wrist/Hands

Hips

Knees

Ankles/Feet

**Please indicate where you primarily experience physical discomfort when performing surgery using robot-assisted laparoscopy:**

Neck

Upper back

Lower back

Shoulders

Elbow

Wrist/Hands

Hips

Knees

Ankles/Feet

**Have you done anything to reduce your physical discomfort?**

No

Yes

Don’t know

**What have you done to reduce your physical discomfort?**

Take microbreaks

Change working posture

Refrain from performing surgery for a period

Take pain-relieving medication

Other
