# Supplementary material for: Ergonomic strain of robotic-assisted versus laparoscopic inguinal hernia repair (ESRALI)—a crossover trial
Source: Surg Endosc. 2025 Mar 31;39(5):3095–105. doi: 10.1007/s00464-025-11676-7 (PMC12041149; doi:10.1007/s00464-025-11676-7)
Supplement: Supplementary file 2 — Supplementary file2 (DOCX 14 KB) [file 464_2025_11676_MOESM2_ESM.docx]

S2. Statistical comparison of muscle activity between cTAPP and rTAPP

| Muscle activity measured by surface EMG (right side) and Wilcoxon test | | | | | | | | | | | | | | |
| --- | --- | --- | --- | --- | --- | --- | --- | --- | --- | --- | --- | --- | --- | --- |
| Muscle | Static activity | | | | | | Median activity | | | | Peak activity | | | |
|  | rTAPP | | cTAPP | | p-value | | rTAPP | | cTAPP | p-value | rTAPP | | cTAPP | p-value |
| AD, mean (SD) | 1.67 (1.40) | | 1.12 (0.54) | | 0.25 | | 8.02 (6.41) | | 5.44 (4.77) | 0.46 | 15.77 (9.01) | | 12.17 (10.84) | 0.38 |
| MD, mean (SD) | 0.98 (0.74) | | 0.92 (0.54) | | 0.64 | | 2.31 (2.31) | | 2.88 (1.96) | 0.46 | 4.57 (2.38) | | 6.79 (2.94) | 0.11 |
| Trap, mean (SD) | 3.70 (3.19) | | 2.66 (0.96) | | 0.64 | | 9.60 (7.31) | | 5.43 (2.64) | 0.016 | 13.45 (8.72) | | 9.51 (4.54) | 0.08 |
| ES, mean (SD) | 5.68 (2.93) | | 5.33 (2.45) | | 0.84 | | 9.33 (2.85) | | 9.69 (4.64) | 1.0 | 14.61 (3.59) | | 16.52 (6.80) | 0.64 |
| Muscle activity measured by surface EMG (left side) and Wilcoxon test | | | | | | | | | | | | | | |
| Muscle | | Static activity | | | | | Median activity | | | | Peak activity | | | |
|  |  | rTAPP | | cTAPP | | p-value | rTAPP | cTAPP | | p-value | rTAPP | cTAPP | | p-value |
| AD, mean (SD) | | 1.10 (0.31) | | 0.87 (0.42) | | 0.38 | 5.67 (3.10) | 3.47 (3.63) | | 0.15 | 11.82 (5.30) | 9.75 (7.47) | | 0.55 |
| MD, mean (SD) | | 1.04 (0.87) | | 0.81 (0.20) | | 0.84 | 1.81 (1.34) | 2.52 (1.99) | | 0.20 | 4.58 (2.47) | 7.46 (4.98) | | 0.46 |
| Trap, mean (SD) | | 4.15 (3.67) | | 2.75 (1.57) | | 0.25 | 9.65 (5.19) | 6.36 (3.68) | | 0.078 | 13.89 (5.84) | 10.79 (4.72) | | 0.078 |
| ES, mean (SD) | | 6.96 (5.82) | | 4.07 (1.60) | | 0.016 | 11.68 (6.79) | 8.22 (5.78) | | 0.023 | 16.03 (7.69) | 14.62 (9.14) | | 0.46 |
| rTAPP = robotic-assisted transabdominal preperitoneal inguinal hernia repair; cTAPP = conventional laparoscopic transabdominal preperitoneal inguinal hernia repair; AD =Anterior deltoid (Musculus deltoideus pars anterior); MD = Middle deltoid (Musculus deltoideus pars medius); Trap = Musculus trapezius; ES = Musculus Erector spinae; SD = Standard deviation | | | | | | | | | | | | | | |
